# Predicting mortality in heart failure: BUN/creatinine ratio in MIMIC-III

**DOI:** 10.3389/fcvm.2025.1510317

**Published:** 2025-03-04

**Authors:** Changsen Zhu, Liyan Wu, Yiyi Xu, Qian Zhang, Wenbo Liu, Yuxiang Zhao, Jun Lyu, Zhuoming Chen

**Affiliations:** ^1^ Department of Rehabilitation Medicine, The First Affiliated Hospital of Jinan University, Guangzhou, Guangdong, China; ^2^ Nursing School, Guangdong Pharmaceutical University, Guangzhou, Guangdong, China; ^3^ Department of Child Rehabilitation, The Ninth Hospital of Xingtai, Xingtai, Hebei, China; ^4^ Clinical Research Department, The First Affiliated Hospital of Jinan University, Guangzhou, Guangdong, China

**Keywords:** heart failure, retrospective cohort, blood nitrogen, creatinine, outcomes

## Abstract

**Aims:**

Heart failure is a critical health issue with high mortality rates. The blood urea nitrogen/creatinine ratio (BCR) has proven more effective at predicting outcomes in heart failure patients than individual assessments of each marker. Nevertheless, the implications of varying BCR levels for outcomes among heart failure patients remain to be fully understood. This study explores the impact of BCR on the outcomes of these patients.

**Methods and results:**

Employing a retrospective cohort design at a single center, this study examined 1,475 heart failure patients from the Medical Information Mart for Intensive Care (MIMIC-III) database, categorized into four quartiles based on their BCR levels. We analyzed survival outcomes using Kaplan–Meier and Cox proportional hazards models, supplemented by restricted cubic splines to elucidate detailed associations. The average age of the patients was 69.52 years, with males constituting 55.6% of the cohort. As BCR values escalated, the average hospital stay increased from 9.64 to 14.15 days, and average survival decreased from 685.11 to 412.68 days. Patients in the highest BCR quartile faced the most severe mortality rates, with 18.8% in-hospital and 78.3% long-term mortality. Nonlinear regression revealed a U-shaped relationship between BCR and mortality: at BCR levels below 12.5, there was no significant correlation with long-term mortality; between 12.5 and 22, BCR appeared to exert a protective effect; and above 22, it emerged as a significant risk factor.

**Conclusions:**

Admission BCR values are non-linearly associated with mortality in heart failure patients, suggesting its utility as a prognostic tool in critical care.

## Introduction

Heart failure (HF) represents a global health challenge characterized by high morbidity and mortality rates. The prevalence of HF has been progressively increasing due to population aging, global population growth, and improved post-diagnosis survival rates (1). This condition imposes substantial societal burdens through escalating healthcare costs and diminished quality of life, while its non-specific early symptoms and signs often lead to delayed diagnosis, ultimately progressing to multiorgan dysfunction or severe complications with high fatality rates (2–4).

Renal impairment constitutes a frequent comorbidity in HF patients and is strongly associated with adverse clinical outcomes (5). While reduced cardiac output and diminished renal perfusion have traditionally been considered primary contributors to HF-associated renal dysfunction, emerging evidence suggests more complex pathophysiological mechanisms involving hemodynamic alterations and neuroendocrine activation. In recent years, the blood urea nitrogen to serum creatinine ratio (BUN/Cr ratio, BCR) has garnered increasing attention as a sensitive biomarker for renal function assessment (6–9).

The dynamic changes in BCR reflect distinct pathophysiological processes in HF patients: (1) Reduced cardiac output activates sympathetic nervous system (SNS) and renin-angiotensin-aldosterone system (RAAS), enhancing sodium and water reabsorption in proximal renal tubules (10). This decreases tubular flow rate and prolongs urea transit time, thereby promoting passive urea reabsorption and BUN elevation (11). (2) Increased antidiuretic hormone (AVP) release upregulates urea transporter UT-A1/3 expression in medullary collecting ducts, further enhancing urea reabsorption (11). (3) Compared to creatinine (Cr), which is primarily excreted through glomerular filtration with minimal extrarenal influences, BUN levels exhibit greater susceptibility to confounding factors including dietary protein intake, hepatic function, and catabolic status (12). These biological characteristics render BCR a more sensitive indicator of renal perfusion alterations than isolated BUN or Cr measurements, particularly in pre-renal azotemia associated with HF.

Multiple studies have demonstrated that elevated BCR strongly correlates with poor prognosis in HF populations. Higher BCR values serve as independent predictors for all-cause mortality and are associated with adverse outcomes in acute HF patients (9, 13). Nevertheless, the relationship between BCR stratification and long-term mortality risk in HF patients remains incompletely elucidated.

Building upon these pathophysiological and clinical foundations, this study aims to evaluate the association between BCR levels and all-cause mortality in HF patients through Kaplan–Meier survival analysis and Cox proportional hazards regression modeling (14). Our findings are expected to provide robust theoretical support for optimizing risk stratification and enhancing prognostic evaluation in HF management.

## Methods

### Data sources

The MIMIC-III database, collaboratively created by Beth Israel Deaconess Medical Center, MIT's Laboratory for Computational Physiology, and Philips Healthcare, encompasses extensive patient information, including demographics, vital signs, laboratory and microbiological tests, radiological diagnoses, observation records, intake and output details, pharmacological treatments, and data regarding hospitalization and survival status, such as discharge or death (15). The MIMIC-III database is a comprehensive, publicly accessible resource that contains information on patients admitted to the intensive care units of a prominent tertiary hospital in Boston, covering the period from June 1, 2001, to October 10, 2012 (16). This study employs version 1.4 of the dataset, analyzing records of over sixty thousand patients treated in the intensive care units at Beth Israel Deaconess Medical Center. This project has received approval from the Institutional Review Boards (IRBs) of Beth Israel Deaconess Medical Center and Massachusetts Institute of Technology. Additionally, the database utilized in this study anonymizes patient information, thereby eliminating the need for informed consent. Our research adheres strictly to the TRIPOD statement; all team members have successfully completed the relevant courses provided by the National Institutes of Health (NIH), thereby earning the necessary certification (Certificate Number: 54878929).

### Population selection

**Figure 1:**
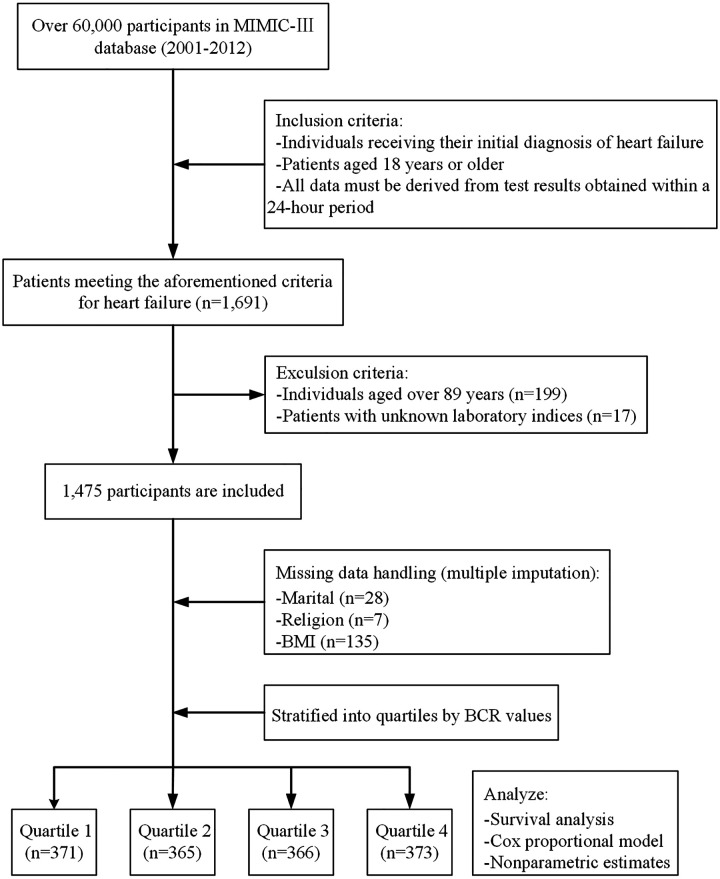
The screening flowchart.

This study initially included 1,691 heart failure patients aged over 18 years from the MIMIC-III database; however, 199 patients who either did not meet the heart failure ICD-9 diagnostic criteria or were older than 89 years, and 17 patients who lacked demographic data and laboratory results within 24 h of intensive care unit (ICU) admission were excluded. Ultimately, 1,475 patients were incorporated into the final study cohort. Based on the quartiles of BCR values within 24 h, patients were stratified into four groups. The data selection process is illustrated in Figure 1.

### Research design

To address the missing values in the data, we employed the multiple imputation method. This approach generates several imputed datasets and combines the results from each to estimate the missing values, thereby enhancing the stability and reliability of the findings. It is particularly suited for handling missing data, as it preserves statistical power to some extent and mitigates biases that may arise from traditional single imputation techniques.

In this study, we utilized the mice package in R for multiple imputation. Specifically, we first extracted samples with missing values under 20% from the database using Structured Query Language (SQL). Then, the mice package was employed to generate 10 imputed datasets, with each dataset undergoing 50 iterations to complete the imputation process. Given that the missing data may follow a Missing At Random (MAR) pattern, we assumed that the missing data is related to the observed variables but not to the missing values themselves. Consequently, a regression-based imputation method was applied. Ultimately, the first complete dataset was utilized for analysis.

Variables included in the analysis encompass (1) demographic characteristics [age, gender, marital status, ethnicity, religious beliefs, insurance status, and Body Mass Index (BMI)], with BMI calculated from weight and height; (2) vital signs [heartrate, Systolic Blood Pressure (SBP), Diastolic Blood Pressure (DBP), Respiratory Rate (RR); Body Temperature (BT), and Saturation of Peripheral Oxygen (SpO2)]; (3) Laboratory test results [Anion Gap, Bicarbonate, Chloride, Glucose, Hematocrit, Hemoglobin, Platelet, Potassium, Sodium, Partial Thromboplastin Time (PTT), International Normalized Ratio (INR), White Blood Cell Count (WBC), and BCR], were all derived from the first measurements taken after admission; (4) other important indices the Simplified Acute Physiology Score II [SAPS II], the Sequential Organ Failure Assessment [SOFA] score, the Elixhauser Comorbidity Index [ECI], and hospital stay duration, determined by the difference between discharge and admission dates. The selection of these variables is based on their widespread use in existing literature and their potential impact on clinical outcomes, such as mortality and disease progression. For instance, BMI and age are recognized as closely associated with patient prognosis, which is why they were included in the analysis model. To control for potential confounding factors, this study incorporates well-established clinical variables such as age, gender, and BMI, while also considering disease severity indicators (e.g., SAPS II, SOFA) and comorbidity burden (e.g., ECI).

The primary outcome of this study is the long-term mortality rate of patients, defined as whether the patient dies within a follow-up period of 90 days or more. Patients in this study were derived from the Metavision and CareVue systems, with follow-up periods of at least 90 days and 4 years, respectively. Based on BCR values, patients were stratified into four quartile groups for survival time analysis. For patients who did not die, survival times were classified based on the system to which they were assigned—either 90 days for Metavision or 4 years for CareVue.

### Statistical analysis

Continuous variables were represented by their means and standard deviations (SD), and differences between groups were compared using Analysis of Variance (ANOVA). Categorical variables were presented as frequencies and percentages, with intergroup differences assessed using the Chi-square or Fisher's exact tests. To evaluate the incidence rate of primary outcome events across different BCR levels, the Kaplan–Meier survival analysis method was employed, and intergroup differences were evaluated using the log-rank test. The Cox proportional hazards model was utilized to estimate the hazard ratios (HRs) and 95% confidence intervals (CIs) between BCR levels and primary outcomes. To validate the proportional hazards assumption, we tested it using Schoenfeld residuals and conducted graphical checks, such as log-minus-log survival plots. The results revealed no significant violations of the assumption, confirming the model's suitability for our data. The analysis was performed using the following adjustment models: Model 1: unadjusted; Model 2: adjusted for age, gender, and ethnicity; Model 3: adjusted for age, gender, ethnicity, marital status, religious beliefs, insurance status, BMI, SAPS II, SOFA, and ECI. The model selection was guided by prior research experience and the need to control for potential confounding factors. In Model 2, adjustments were made for basic demographic characteristics (such as age, gender, and ethnicity), while Model 3 further adjusted for disease severity (e.g., SAPS II, SOFA) and comorbidity burden (e.g., ECI) to mitigate the potential influence of these factors on the outcomes. Restricted cubic splines and smooth curve fitting methods (penalized splines) were employed to examine the association between the BCR index and outcomes.

Subgroup analyses aimed to examine the relationship between mortality rates and age (<65, ≥65), gender, ethnicity, BMI (<25, 25–29.99, ≥30), SAPS II (<40, ≥40), SOFA (≤2, >2), and ECI (≤11, >11). The *p*-values for interactions among subgroups and across various categories were calculated using the log-likelihood ratio test.

All analyses were performed using Navicat Premium (version 15.0.23) and R software (version 4.2.3), with missing data being imputed using the “mice” package, and Kaplan–Meier survival analysis and Cox proportional hazards regression models were conducted using the “survival” package. A two-sided *p*-value of less than 0.05 was deemed statistically significant.

## Results

### Baseline data

In this study, three variables—marital status, religious beliefs, and BMI—had missing data. Details on the specific missing values can be found in Table 1.

**Table 1:** Missing variables.

Variables	Number of missing data	Percentage of missing data (%)
Marital	28	1.90
Religion	7	0.47
BMI	135	9.15

BMI, body mass index.

This study ultimately included 1,475 heart failure patients who met the inclusion criteria. The average age of the enrolled patients was 69.52 years, comprising 820 males (55.6%). The average BCR for all patients was 23.21.

Patients were stratified into four quartiles based on their BCR values: Quartile 1 (BCR < 16.7) comprised 371 individuals; Quartile 2 (16.7 ≤ BCR < 22.0) included 365; Quartile 3 (22.0 ≤ BCR < 28.0) contained 366; and Quartile 4 (BCR ≥ 28.0) encompassed 373 individuals. Significant differences were observed across the quartiles regarding ethnicity, marital status, religious beliefs, and insurance coverage. The highest BCR quartile had the largest proportion of white individuals, the lowest BCR quartile had the highest proportion of black individuals, and the third quartile had the highest percentage of married individuals. Moreover, there were significant differences in vital signs among the four groups; as the BCR increased, the average SBP decreased from 143.65 to 137.35, and the average DBP decreased from 81.93 to 78.22. In terms of laboratory tests, variations were observed in Anion Gap, Bicarbonate, Chloride, Hematocrit, Hemoglobin, Platelet, Potassium, Sodium, and WBC across the four groups. Further details are provided in Table 2.

**Table 2:** Baseline characteristics.

Variables	BCR				
	<16.7	≥16.7, <22.0	≥22.0, <28.0	≥28.0	*p*-value
*N* = 1,475	371	365	366	373	
Age, mean (SD)	70.16 (12.70)	68.99 (14.00)	69.67 (13.15)	69.27 (13.55)	0.659
Gender, *n* (%)					0.128
Male (820)	198 (53.4)	218 (59.7)	210 (57.4)	194 (52.0)	
Female (655)	173 (46.6)	147 (40.3)	156 (42.6)	179 (48.0)	
Ethnicity, *n* (%)					<0.001
White (1,027)	204 (55.0)	250 (68.5)	277 (75.7)	296 (79.4)	
Black (226)	114 (30.7)	55 (15.1)	29 (7.9)	28 (7.5)	
Other (222)	53 (14.3)	60 (16.4)	60 (16.4)	49 (13.1)	
Marital, *n* (%)					0.001
Married (1,168)	281 (75.7)	278 (76.2)	312 (85.2)	297 (79.6)	
Unmarried (296)	89 (24.0)	87 (23.8)	50 (13.7)	70 (18.8)	
Other (11)	1 (0.3)	0 (0.0)	4 (1.1)	6 (1.6)	
Religion, *n* (%)					0.043
Christian (927)	251 (67.7)	229 (62.7)	227 (62.0)	220 (59.0)	
Jewish (219)	44 (11.9)	46 (12.6)	56 (15.3)	73 (19.6)	
Other (329)	76 (20.5)	90 (24.7)	83 (22.7)	80 (21.4)	
Insurance, *n* (%)					0.007
Government (1,241)	300 (80.9)	292 (80.0)	324 (88.5)	325 (87.1)	
Private (229)	69 (18.6)	72 (19.7)	40 (10.9)	48 (12.9)	
Self pay (5)	2 (0.5)	1 (0.3)	2 (0.5)	0 (0.0)	
BMI, mean (SD)	29.74 (8.02)	30.48 (9.79)	29.79 (9.01)	29.55 (8.19)	0.503
SAPS II, mean (SD)	38.33 (12.59)	38.20 (12.27)	38.83 (12.78)	37.87 (12.09)	0.769
SOFA, mean (SD)	4.76 (2.96)	4.83 (2.76)	4.80 (2.82)	4.62 (2.56)	0.748
ECI, mean (SD)	11.70 (7.47)	11.47 (8.44)	10.84 (7.37)	12.22 (8.29)	0.120
Heartrate, mean (SD)	98.38 (19.32)	99.26 (19.54)	99.17 (20.74)	99.03 (20.63)	0.932
SBP, mean (SD)	143.65 (23.29)	142.51 (26.50)	142.26 (24.69)	137.35 (22.97)	0.002
DBP, mean (SD)	81.93 (16.62)	80.86 (18.77)	81.10 (19.49)	78.22 (16.95)	0.031
RR, mean (SD)	28.86 (6.77)	28.27 (6.08)	28.74 (6.28)	28.50 (6.53)	0.601
BT, mean (SD)	37.22 (0.78)	37.21 (0.78)	37.17 (0.80)	37.21 (0.72)	0.805
SpO2, mean (SD)	99.46 (1.25)	99.40 (1.45)	99.44 (1.30)	99.38 (1.18)	0.857
Anion gap, mean (SD)	17.05 (4.27)	16.91 (4.93)	16.77 (4.41)	15.89 (3.98)	0.001
Bicarbonate, mean (SD)	26.96 (4.73)	27.32 (5.68)	27.38 (5.31)	29.35 (6.07)	<0.001
Chloride, mean (SD)	102.74 (5.89)	103.07 (6.34)	102.13 (6.48)	99.75 (7.12)	<0.001
Glucose, mean (SD)	185.60 (100.41)	178.36 (86.85)	183.59 (80.40)	174.27 (68.97)	0.255
Hematocrit, mean (SD)	35.53 (6.10)	35.41 (5.65)	35.06 (5.61)	34.33 (5.82)	0.022
Hemoglobin, mean (SD)	11.48 (2.14)	11.59 (1.93)	11.48 (1.87)	11.17 (2.01)	0.028
Platelet, mean (SD)	273.84 (133.96)	239.04 (113.51)	249.19 (125.47)	246.75 (109.66)	0.001
Potassium, mean (SD)	5.05 (1.11)	4.88 (1.02)	4.81 (0.90)	4.82 (0.96)	0.004
Sodium, mean (SD)	139.33 (4.07)	139.33 (4.38)	138.75 (4.56)	138.20 (5.66)	0.002
PTT, mean (SD)	49.66 (33.46)	53.54 (36.17)	50.10 (32.30)	49.29 (32.73)	0.294
INR, mean (SD)	2.08 (2.09)	1.93 (1.29)	2.02 (2.38)	2.11 (1.85)	0.624
WBC, mean (SD)	12.83 (9.00)	11.24 (5.37)	12.30 (6.48)	11.60 (5.76)	0.007

BCR, blood urea nitrogen/creatinine ratio; SD, standard deviations; BMI, body mass index; SAPS II, simplified acute physiology score II; SOFA, sequential organ failure assessment; ECI, elixhauser comorbidity index; SBP, systolic blood pressure; DBP, diastolic blood pressure, RR, respiratory rate; BT, body temperature; SPO2, saturation of peripheral oxygen; PTT, partial thromboplastin time; INR, international normalized ratio; WBC, white blood cell count.

### Outcomes

The study primarily focused on long-term mortality rates. As indicated in Table 3, significant variations were observed among the groups regarding in-hospital duration, survival time, in-hospital mortality, and long-term mortality rates. The overall in-hospital mortality rate stood at 12.7%, with a long-term mortality rate of 62.9%. As BCR values increased, the average hospital stay was extended from 9.64 to 14.15 days, while the average survival period was reduced from 685.11 to 412.68 days. The group with the highest BCR values exhibited the highest rates of in-hospital and long-term mortality, at 18.8% and 78.3%, respectively. In contrast, the group with BCR values in the second quartile demonstrated the lowest mortality rates, at 8.8% and 51.2%, respectively.

**Table 3:** Outcomes of patients.

Variables	BCR				
	<16.7	≥16.7, <22.0	≥22.0, <28.0	≥28.0	*p*-value
*N* = 1,475	371	365	366	373	
Hospital stay	9.64	10.85	11.58	14.15 (11.80)	<0.001
Mean (SD)	(8.97)	(9.35)	(9.69)		
Survival time	685.11	615.36	524.26	412.68	<0.001
Mean (SD)	(721.04)	(722.38)	(706.52)	(691.51)	
Inhospital status, *n* (%)					<0.001
Survival (1,287)	333 (89.8)	333 (91.2)	318 (86.9)	303 (81.2)	
Mortality (188)	38 (10.2)	32 (8.8)	48 (13.1)	70 (18.8)	
Long-term status, *n* (%)					<0.001
Survival (547)	157 (42.3)	178 (48.8)	131 (35.8)	81 (21.7)	
Mortality (928)	214 (57.7)	187 (51.2)	235 (64.2)	292(78.3)	

BCR, blood nitrogen/creatinine ratio; SD, standard deviations.

### BCR and mortality rate

**Figure 2:**
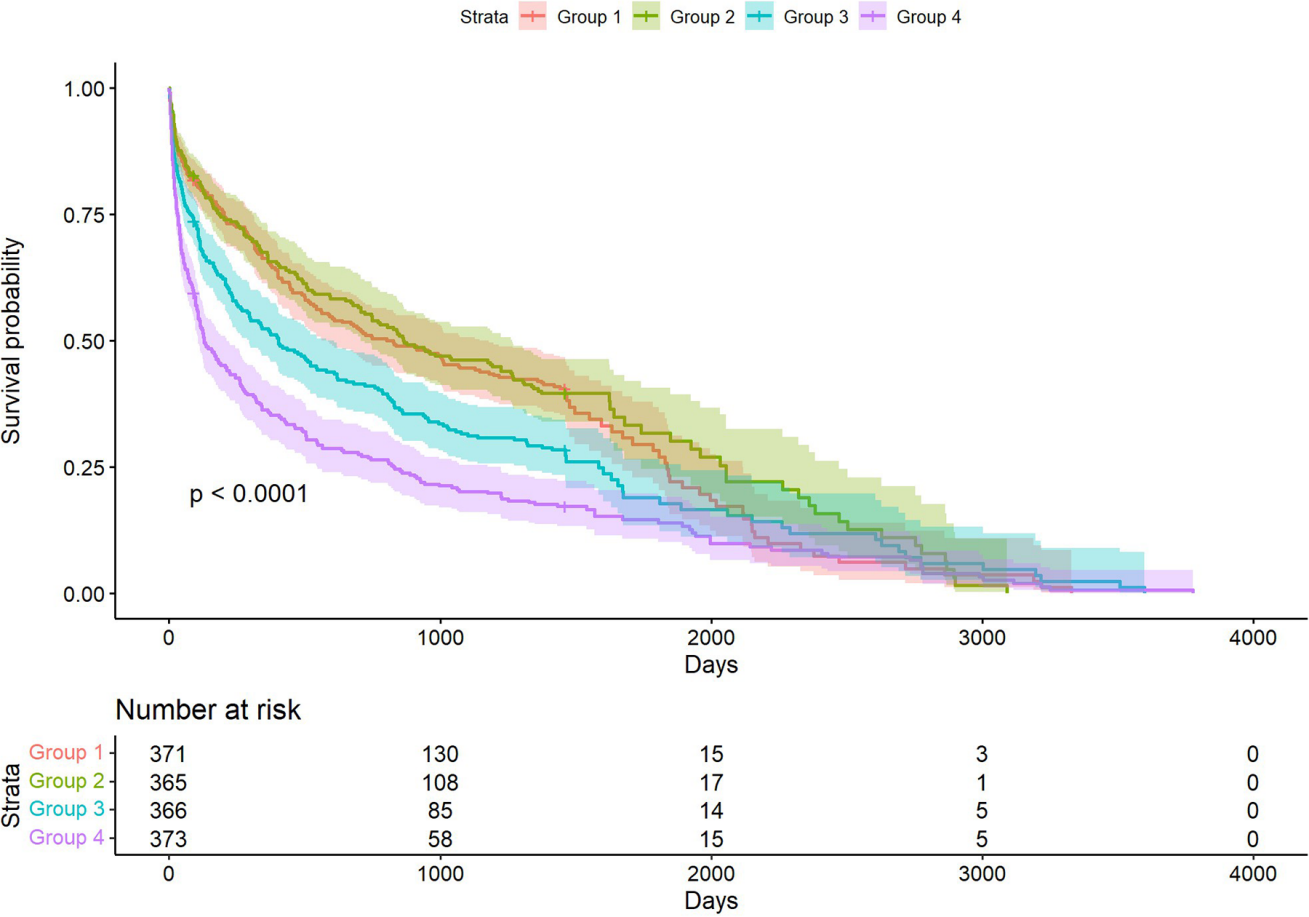
Kaplan–Meier curves depicting long-term mortality across groups.

The Kaplan–Meier survival curves illustrating the incidence of primary outcomes across BCR quartiles are shown in Figure 2. Significant statistical differences in long-term mortality rates were observed among the groups (*P* < 0.001).

By constructing three Cox regression models to analyze the independent relationship between BCR and mortality risk, BCR, as a continuous variable, showed a significant correlation with the patient's long-term all-cause mortality rate in both the unadjusted Model 1 (HR 1.02; 95% CI 1.01–1.03; *P* < 0.001) and the fully adjusted Model 3 (HR 1.02; 95% CI 1.01–1.12; *P* < 0.001). When BCR was categorized into quartiles, the fully adjusted Model 3, using Quartile 1 as the reference, indicated a potential nonlinear association with outcome variables: Quartile 2 showed a hazard ratio (HR) of 0.98 (95% CI 0.80–1.19, *P* = 0.826); Quartile 3, an HR of 1.28 (95% CI 1.06–1.55, *P* = 0.012); and Quartile 4, an HR of 1.80 (95% CI 1.50–2.17, *P* < 0.001).

Categorizing BCR into tertiles and quintiles produces significantly different results, as illustrated in Table 4. Given the varied outcomes from these categorizations, it is inferred that the relationship between BCR and mortality is not linear.

**Table 4:** HRs (95% CIs) for all-cause mortality across groups.

Variables	Model 1		Model 2		Model 3	
	HR(95%CI)	*p*-value	HR(95%CI)	*p*-value	HR(95%CI)	*p*-value
BCR	1.02 (1.01, 1.03)	<0.001	1.02 (1.01, 1.03)	<0.001	1.02 (1.01, 1.12)	<0.001
Tertiles						
<18.3	Reference		Reference		Reference	
≥18.3, <25.3	1.15 (0.97, 1.36)	0.110	1.13 (0.96, 1.34)	0.150	1.15 (0.97, 1.36)	0.112
≥25.3	1.79 (1.53, 2.10)	<0.001	1.81 (1.54, 2.15)	<0.001	1.71 (1.45, 2.02)	<0.001
Quartiles						
<16.7	Reference		Reference		Reference	
≥16.7, <22.0	0.95 (0.78, 1.16)	0.633	0.94 (0.77, 1.14)	0.511	0.98 (0.80, 1.19)	0.826
≥22.0, <28.0	1.31 (1.09, 1.58)	0.004	1.31 (1.08, 1.58)	0.006	1.28 (1.06, 1.55)	0.012
≥28.0	1.86 (1.55, 2.22)	<0.001	1.88 (1.56, 2.26)	<0.001	1.80 (1.50, 2.17)	<0.001
Quintiles						
<15.3	Reference		Reference		Reference	
≥15.3, <20.0	1.00 (0.81, 1.25)	0.990	0.98 (0.78, 1.22)	.837	1.01 (0.81, 1.26)	0.907
≥20.0, <23.8	1.09 (0.88, 1.36)	0.432	1.06 (0.85, 1.32)	0.593	1.12 (0.90, 1.40)	0.317
≥23.8, <30.0	1.40 (1.14, 1.72)	0.001	1.40 (1.13, 1.74)	0.002	1.37 (1.11, 1.69)	0.004
≥30.0	1.83 (1.50, 2.23)	<0.001	1.83 (1.49, 2.26)	<0.001	1.79 (1.46, 2.21)	<0.001

BCR, blood urea nitrogen/creatinine ratio.

Cox proportional hazards regression models were used to calculate hazard ratios (HRs) with 95% confidence intervals (CIs).

Model 1 was unadjusted.

Model 2 was adjusted for age, gender, and ethnicity.

Model 3 was adjusted for age, gender, ethnicity, marital, religion, insurance, BMI, SAPS II, SOFA, and ECI.

### Nonlinear relationship assessment

**Figure 3:**
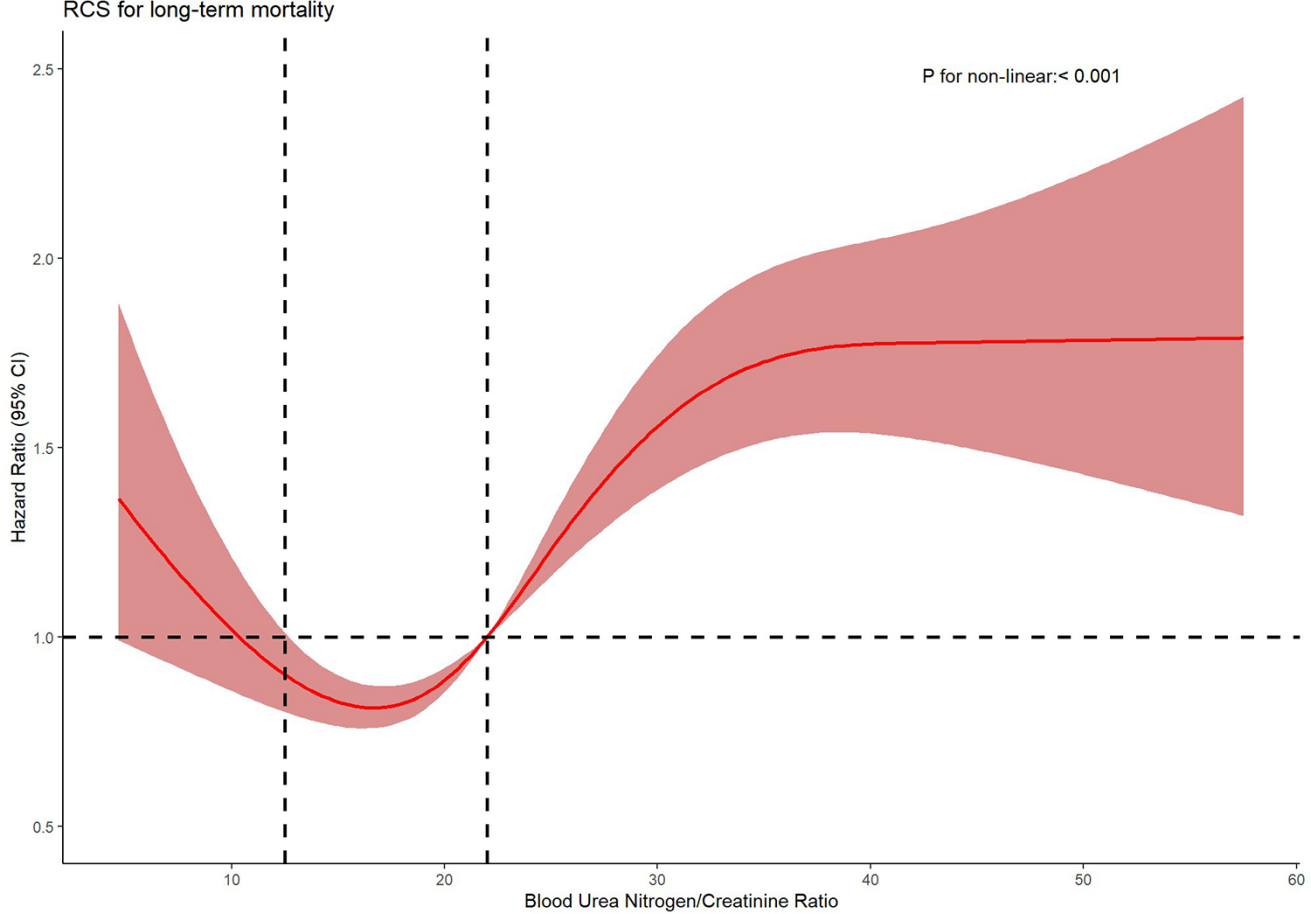
Nonparametric estimates of all-cause mortality on blood urea nitrogen/creatinine ratio among patients with heart failure.

Further analysis using Cox proportional hazards regression models with restricted cubic splines and penalized splines confirmed a significant nonlinear relationship between BCR and all-cause mortality (*P* < 0.001), as depicted in Figure 3. Specifically, this U-shaped curve can be delineated into three distinct segments: below 12.5, BCR exhibits no significant correlation with long-term mortality in heart failure patients; between 12.5 and 22, it demonstrates a protective effect; above 22, it emerges as a risk factor.

### Subgroup analysis

Subgroup analyses that explored the interplay between various risk factor levels and long-term all-cause mortality in heart failure patients revealed varying hazard ratios and corresponding *p*-values across the groups (Table 5). Using Quartile 1 (BCR < 16.7) as the reference, the hazard ratios for subjects in Quartile 4 (BCR ≥ 28.0) exceeded 1 regardless of the factor-based reclassification within each subgroup (*p* < 0.05), suggesting that the formation of subgroups did not influence the outcomes. In Quartile 3 (22.0 ≤ BCR < 28.0), subjects aged ≥65, male gender, Caucasian ethnicity, a BMI of 25–29.99, SAPS II <40, SOFA >2, and an ECI ≤11 exhibited hazard ratios greater than 1 (*p* < 0.05), indicating a higher mortality risk compared to other subgroups. Meanwhile, in Quartile 2 (16.7 ≤ BCR < 22.0), Black subjects had a hazard ratio of 0.47 (*p* = 0.006), signifying a lower mortality risk than in other subgroups. However, the interaction tests' *p*-values all exceeded 0.05, indicating that other factors had no significant effect on the long-term mortality risk among heart failure patients.

**Table 5:** Subgroup analysis of relationship between groups and all-cause mortality.

Variables	BCR							
	<16.7	≥16.7, <22.0		≥22.0, <28.0		≥28.0		p-interaction
		HR(95%CI)	*p*-value	HR(95%CI)	*p*-value	HR(95%CI)	*p*-value	
Age								0.831
<65 (450)	Reference	1.03 (0.73, 1.45)	0.851	1.39 (0.98, 1.98)	0.063	1.93 (1.40, 2.67)	<0.001	
≥65 (1,025)	Reference	0.91 (0.72, 1.16)	0.463	1.30 (1.04, 1.62)	0.021	1.82 (1.47, 2.26)	<0.001	
Gender								0.668
Male (820)	Reference	0.93 (0.72, 1.20)	0.572	1.35 (1.06, 1.73)	0.015	2.02 (1.59, 2.57)	<0.001	
Female (655)	Reference	0.98 (0.72, 1.33)	0.885	1.26 (0.95, 1.68)	0.109	1.72 (1.32, 2.25)	<0.001	
Ethnicity, *n* (%)								0.145
White (1,027)	Reference	1.09 (0.86, 1.40)	0.477	1.33 (1.06, 1.68)	0.015	1.93 (1.55, 2.41)	<0.001	
Black (226)	Reference	0.47 (0.27, 0.80)	0.006	1.43 (0.86, 2.38)	0.174	2.30 (1.33, 3.97)	0.003	
Other (222)	Reference	1.00 (0.59, 1.69)	0.994	1.36 (0.83, 2.24)	0.228	1.72 (1.04, 2.85)	0.034	
BMI								0.992
<25 (446)	Reference	1.00 (0.69, 1.44)	0.984	1.19 (0.84, 1.69)	0.317	1.80 (1.30, 2.51)	<0.001	
25–29.99 (456)	Reference	0.88 (0.61, 1.28)	0.506	1.52 (1.09, 2.12)	0.013	2.49 (1.80, 3.45)	<0.001	
≥30 (573)	Reference	0.97 (0.72, 1.31)	0.852	1.26 (0.93, 1.70)	0.134	1.55 (1.16, 2.07)	0.003	
SAPS II								0.887
<40 (861)	Reference	0.96 (0.74, 1.25)	0.753	1.31 (1.02, 1.68)	0.035	1.97 (1.56, 2.49)	<0.001	
≥40 (614)	Reference	0.93 (0.69, 1.26)	0.291	1.30 (0.98, 1.72)	0.066	1.70 (1.29, 2.24)	<0.001	
SOFA								0.764
≤2 (319)	Reference	0.99 (0.64, 1.53)	0.961	1.34 (0.90, 2.01)	0.148	2.22 (1.52, 3.25)	<0.001	
>2 (1,156)	Reference	0.94 (0.75, 1.17)	0.568	1.30 (1.06, 1.61)	0.014	1.77 (1.45, 2.17)	<0.001	
ECI								0.107
≤11 (754)	Reference	1.20 (0.90, 1.60)	0.218	1.81 (1.38, 2.38)	<0.001	2.32 (1.77, 3.02)	<0.001	
>11 (721)	Reference	0.82 (0.63, 1.08)	0.401	0.98 (0.75, 1.28)	0.883	1.58 (1.23, 2.01)	<0.001	

BCR, blood urea nitrogen/creatinine ratio; BMI, body mass index; SAPS II, simplified acute physiology score II; SOFA, sequential organ failure assessment; ECI, elixhauser comorbidity index.

Although the results of the subgroup analysis offer some insights into the risks for specific populations, the relatively small sample sizes in certain subgroups may introduce some instability in the findings. Therefore, we should interpret these results with caution, avoiding overgeneralization.

## Discussion

This study, utilizing restricted cubic spline models, is the first to identify a nonlinear association between the blood urea nitrogen-to-creatinine ratio (BCR) and all-cause mortality in patients with chronic heart failure. This finding indicates that the relationship between BCR and mortality is not a simple linear increase or decrease but exhibits distinct trends across different BCR levels, collectively forming a U-shaped curve.

### Detailed analysis

1. BCR < 12.5: Within this range, no significant association was observed between BCR and all-cause mortality. This suggests that at lower BCR levels, its impact on mortality risk in heart failure patients may be negligible, or confounding factors (e.g., malnutrition, cachexia, or dilutional effects from volume overload) dominate mortality outcomes, overshadowing the prognostic role of BCR.
2. BCR 12.5–22: A hazard ratio (HR) < 1 within this interval implies a protective effect of BCR, potentially reflecting an adaptive equilibrium between renal perfusion pressure and metabolic demands. This balance may stabilize cardiorenal homeostasis, thereby reducing mortality risk. The neutral-to-protective association in this range aligns with preserved neurohormonal activation (e.g., moderate RAAS activity) that sustains circulatory compensation without inducing overt renal congestion or hypoperfusion.
3. BCR > 22: An HR > 1 signifies that elevated BCR levels correlate with increased all-cause mortality. A BCR exceeding 22 characterizes disproportionate urea retention secondary to renal hypoperfusion, a hallmark of type 1 cardiorenal syndrome. This threshold corresponds to neurohormonal hyperactivation in decompensated heart failure, where angiotensin II-driven renal vasoconstriction and urea transporter upregulation (e.g., UT-A1/3 in collecting ducts) exacerbate urea reabsorption [23]. The elevated BCR reflects progressive renal dysfunction and worsening cardiac output, ultimately contributing to higher long-term mortality.

### BUN and creatinine

Creatinine is a by-product of muscle metabolism, produced consistently in skeletal muscle. After being filtered through the glomeruli, creatinine appears in urine and is actively secreted in the renal tubules, which can lead to an overestimation of the glomerular filtration rate (GFR) when using creatinine clearance rates. High creatinine may indicate impaired kidney function, and kidney damage can lead to the accumulation of toxins, which can further damage cardiomyocytes; it may also lead to an increased cardiac load, which in turn can cause cardiomyocyte oedema, necrosis, etc., inducing or exacerbating heart failure (17, 18). Creatinine levels are influenced by various factors, such as muscle mass, which might not accurately reflect renal function (12). Blood urea nitrogen, a metabolic by-product of protein metabolism, is closely associated with the incidence and mortality of heart failure (19, 20). BUN levels, influenced by protein intake, catabolism, and renal tubular reabsorption, reflect the severity of both renal function and heart failure. Compared to serum creatinine, BUN is more significantly affected by nutritional status and catabolic metabolism (21). However, as it is a product of protein breakdown, it is not affected by muscle mass (22). The dynamic interaction between heart failure and renal function, along with BUN levels, may reflect “vasoconstrictive nephropathy” which is associated with neurohormonal activation in heart failure patients (23).

### BCR as a prognostic marker for heart failure

In chronic heart failure patients, renal salt and water homeostasis alterations are primarily driven by activation of the renin-angiotensin-aldosterone system and the non-osmotic release of arginine vasopressin (24). The glomerular filtration rate is regulated by the pressure differential between the afferent and efferent arterioles. Compared to the GFR, BCR may serve as a more suitable indicator for assessing the effective circulating volume in heart failure (25).

Recent studies have highlighted the prognostic significance of the BUN/Cr ratio in heart failure (26). Higher BUN/Cr ratios have been associated with increased mortality, potentially due to their reflection of neurohormonal activation and hemodynamic changes. Research has indicated that an elevated BUN/Cr ratio may signify greater renal hypoperfusion and more severe volume overload, both of which contribute to worse outcomes in heart failure patients. Additionally, some studies suggest that changes in the BUN/Cr ratio over time may provide additional prognostic value beyond a single measurement at admission. For instance, research on patients with acute ischemic stroke has shown that a decreasing BUN/Cr ratio over 24–72 h correlates with improved neurological outcomes, suggesting that dynamic monitoring of this ratio may enhance risk stratification (27).

By employing the method of restricted curves, we discovered that there is a non—linear correlation between BCR and the mortality rate of heart failure patients, and we identified the specific intervals with significant associations. This finding differs from previous studies. Some scholars used the ROC curve to assess the predictive ability of BCR for the all—cause mortality rate in chronic heart failure patients, obtaining a cutoff value of 19.37, and then divided the BCR values into two groups for analysis (28). However, our research method and results provide a more nuanced relationship between BCR and heart failure prognosis, which can help clinicians more accurately assess patients' conditions.

Relevant studies have shown that the BUN/CR ratio can enhance the ability to predict the decline of glomerular filtration rate (GFR), which is of great significance for the treatment and management of heart failure patients. Accurate prediction of GFR changes can help doctors adjust treatment plans in a timely manner, strengthen the monitoring of patients, and design future intervention measures (29). However, there are also certain differences among different research results. For instance, some studies have found that a high BUN/Cr value is associated with the mortality rate of acute heart failure, while a low BCR has no statistical significance in relation to acute heart failure (10, 30, 31). In addition, among heart failure patients regularly followed up in clinics, there is a significant difference between the urea nitrogen/creatinine value and the adverse outcome of heart failure (25). Compared with these studies, our research not only verified the correlation between BCR and heart failure prognosis, but also further clarified the relationship between the specific range of BCR and heart failure. These specific values provide more directive references for clinical application, enabling doctors to more accurately assess the prognosis according to the changes in patients' BCR values, adjust treatment measures in a timely manner, and thus improve the final outcome of heart failure patients.

### Clinical significance of research results

The discovery of this non-linear association has provided new quantitative evidence for the risk stratification management of heart failure patients. In clinical practice, doctors can more accurately assess the risk of death based on the BCR value of patients. They can adopt different treatment strategies and monitoring measures for heart failure patients in different BCR ranges. For example, for high—risk patients with BCR > 22, more active intervention and close monitoring are needed to reduce their risk of death; for patients with BCR between 12.5 and 22, the existing treatment plan can be maintained to a certain extent while paying attention to the changes in the condition; and for patients with BCR < 12.5, regular monitoring of BCR changes is also needed to timely detect potential risk factors.

### Research significance

Diagnosing early-stage heart failure is challenging and represents a primary cause for the high treatment and hospitalization costs associated with later stages of the disease (32, 33). This imposes a significant economic burden on society, especially on low- and middle-income countries, which bear approximately 80% of the global cardiovascular disease burden (34). Despite the availability of heart failure treatment guidelines, the prognosis for patients remains poor, with a five-year mortality rate exceeding 50% in most cases. Therefore, secondary prevention has become crucial in managing heart failure, underscoring the importance of early detection and elevating the search for biomarkers to a prominent research topic (35–39).

Traditionally, GFR measurements have relied on specific markers such as inulin clearance, which are both time-consuming and costly, making them impractical for routine clinical use. By contrast, measurements of serum creatinine and blood urea nitrogen are more accessible and affordable, providing a viable alternative for resource-poor settings or critically ill patients.

According to our findings, a specific range of elevated blood urea nitrogen and creatinine ratios at admission may signify an adverse clinical phenotype in heart failure patients. However, the BCR is not an optimal indicator of renal urea handling, as it is highly susceptible to non-renal factors such as diet and protein catabolism. In recent years, new renal biomarkers, such as Neutrophil Gelatinase-Associated Lipocalin (NGAL), have demonstrated high specificity in detecting acute kidney injury. This suggests a plausible hypothesis that results obtained with the less-specific renal indicator BCR could be further refined by employing these highly specific renal biomarkers, potentially yielding more accurate prognostic reference values. This approach could potentially represent a future research direction for enhancing diagnostic and management strategies in heart failure.

Our study also reveals compelling findings: as the blood urea nitrogen/creatinine ratio increases, the duration of hospital stays for heart failure patients lengthens, and their average survival time decreases. The relationship between hospital stay duration and all-cause mortality rates in heart failure patients warrants further investigation.

### Limitations

The limitations of this study are primarily reflected in the following aspects. First, the data were sourced from the MIMIC-III database, which predominantly includes samples from a Caucasian population, potentially leading to selection bias and affecting the external validity of the findings. Heart failure patients from different racial and regional backgrounds may exhibit variations in clinical characteristics, disease progression, and treatment responses. Therefore, the generalizability of the results should be verified through more diverse datasets in future studies, which should incorporate broader racial and geographic backgrounds to enhance the applicability of the findings.

Second, our analysis was based solely on BUN/Cr ratios measured within the first 24 h of ICU admission, without considering their dynamic changes during hospitalization. However, the BUN/Cr ratio in heart failure patients may fluctuate as the disease progresses, thus future research should include continuous monitoring data throughout hospitalization to more accurately assess the impact of BUN/Cr ratios on prognosis.

Additionally, the MIMIC-III database does not include lifestyle factors (such as diet, exercise, etc.), which could significantly influence BUN and creatinine levels, and in turn, patient outcomes. Future studies should aim to integrate these variables or explore other databases that capture such data to further enhance the comprehensiveness of the research.

Lastly, although missing data were handled (e.g., excluding variables with more than 20% missing values), this approach may introduce selection bias, potentially affecting the accuracy of the results.

Overall, these limitations emphasize the need for cautious interpretation of our findings and suggest that future research should further refine study designs by incorporating more diverse samples, continuous data collection, and improved data handling methods.

## Conclusion

Our study revealed a non-linear correlation between BCR and long-term mortality. Physicians can more accurately assess a patien's mortality risk based on different ranges of BCR values, allowing for the formulation of personalized treatment strategies. It is advisable to maintain the patient's BCR within the range of 12.5–22 to optimize long-term survival rates.

## Data Availability

The datasets presented in this study can be found in online repositories. The names of the repository/repositories and accession number(s) can be found in the article/Supplementary Material.
