# Supplementary material for: Predicting mortality in heart failure: BUN/creatinine ratio in MIMIC-III
Source: Front Cardiovasc Med. 2025 Mar 4;12:1510317. doi: 10.3389/fcvm.2025.1510317 (PMC11913865; doi:10.3389/fcvm.2025.1510317)
Supplement: Supplementary file 2 [file Datasheet2.docx]

mydata<- read.csv ("heart_final - bcr_max.csv")

str(mydata)

mydata$group <- factor(mydata$group, labels = c('Group 1', 'Group 2','Group 3','Group 4'))

mydata$group3 <- factor(mydata$group3, labels = c('Group 1', 'Group 2','Group 3'))

mydata$group5 <- factor(mydata$group5, labels = c('Group 1', 'Group 2','Group 3','Group 4','Group 5'))

mydata$gender <- factor(mydata$gender,labels=c('Male','Female'))

mydata$ethnicity <- factor(mydata$ethnicity,labels=c('White','Black','Other'))

mydata$marital <- factor(mydata$marital,labels=c('Married','Unmarried','Other'))

mydata$religion <- factor(mydata$religion,labels=c('Christian','Jewish','Other'))

mydata$insurance <- factor(mydata$insurance,labels=c('Government','Private','Self Pay'))

str(mydata)

attach(mydata)

library(tableone)

myvars <- c("Age", "BMI", "SAPSII", "SOFA", "heartrate_max",

"sysbp_max", "diasbp_max", "meanbp_max", "resprate_max", "tempc_max",

"spo2_max", "AnionGap_max", "Bicarbonate_max", "chloride_max", "Glucose_max",

"hematocrit_max", "hemoglobin_max", "platelet_max", "Potassium_max", "PTT_max",

"INR_max", "Sodium_max", "WBC_max", "elixhauser_vanwalraven",

"Length.of.hospital.stay", "month",

"gender", "ethnicity", "marital", "religion", "insurance",

"Inhospital.status", "status")

catvars <- c("gender", "ethnicity", "marital", "religion", "insurance",

"Inhospital.status", "status")

tab1 <- CreateTableOne(vars = myvars, data = mydata, factorVars = catvars)

table1<-print(tab1,showAllLevels = TRUE)

write.csv(table1,"zongti.csv")

tab2 <- CreateTableOne(vars = myvars, strata = "group", data = mydata, factorVars = catvars)

table2 <- print(tab2, showAllLevels = TRUE)

write.csv(table2,"fenzu.csv")

# figure 2

library(survival)

library(survminer)

surv_obj <- with(mydata, Surv(month, status))

km_fit <- survfit(surv_obj ~ group, data = mydata)

ggsurvplot(km_fit, data = mydata, mark.time = TRUE, conf.int = TRUE, xlab = "Days", pval = TRUE, legend.labs = c("Group 1", "Group 2","Group 3", "Group 4"), risk.table = TRUE)

survdiff_result <- survdiff(surv_obj ~ group, data = mydata)

print(survdiff_result)

fit <- coxph(Surv(month, status) ~ group, data = mydata)

summary(fit)

# adjusted for age, gender, ethnicity

fit <- coxph(Surv(month, status) ~ group + Age + gender + ethnicity , data = mydata)

summary(fit)

# adjusted for age, gender, ethnicity, marital, religion, insurance, BMI, SAPS II, SOFA, and ECI

fit <- coxph(Surv(month, status) ~ group + Age + gender + ethnicity + marital + religion + insurance + BMI + SAPSII + SOFA + elixhauser_vanwalraven , data = mydata)

summary(fit)

fit <- coxph(Surv(month, status) ~ bcr_max, data = mydata)

summary(fit)

# adjusted for age, gender, ethnicity(bcr_max)

fit <- coxph(Surv(month, status) ~ bcr_max + Age + gender + ethnicity , data = mydata)

summary(fit)

# adjusted for age, gender, ethnicity, marital, religion, insurance, BMI, SAPS II, SOFA, and ECI(bcr_max)

fit <- coxph(Surv(month, status) ~ bcr_max + Age + gender + ethnicity + marital + religion + insurance + BMI + SAPSII + SOFA + elixhauser_vanwalraven , data = mydata)

summary(fit)

fit <- coxph(Surv(month, status) ~ group3, data = mydata)

summary(fit)

# adjusted for age, gender, ethnicity(group3)

fit <- coxph(Surv(month, status) ~ group3 + Age + gender + ethnicity , data = mydata)

summary(fit)

# adjusted for age, gender, ethnicity, marital, religion, insurance, BMI, SAPS II, SOFA, and ECI(group3)

fit <- coxph(Surv(month, status) ~ group3 + Age + gender + ethnicity + marital + religion + insurance + BMI + SAPSII + SOFA + elixhauser_vanwalraven , data = mydata)

summary(fit)

fit <- coxph(Surv(month, status) ~ group5, data = mydata)

summary(fit)

# adjusted for age, gender, ethnicity(group5)

fit <- coxph(Surv(month, status) ~ group5 + Age + gender + ethnicity , data = mydata)

summary(fit)

# adjusted for age, gender, ethnicity, marital, religion, insurance, BMI, SAPS II, SOFA, and ECI(group5)

fit <- coxph(Surv(month, status) ~ group5 + Age + gender + ethnicity + marital + religion + insurance + BMI + SAPSII + SOFA + elixhauser_vanwalraven , data = mydata)

summary(fit)

library(survival)

library(rms)

library(ggplot2)

mydata <- na.omit(mydata)

dd <- datadist(mydata)

options(datadist='dd')

fit_inhospital <- cph(Surv(month, status) ~

rcs(bcr_max, 4), data=mydata)

OR <- Predict(fit_inhospital, bcr_max, fun=exp, ref.zero = TRUE)

OR

anova_fit_inhospital <- anova(fit_inhospital)

print(anova_fit_inhospital)

HR_inhospital <- Predict(fit_inhospital, bcr_max, fun=exp, ref.zero = TRUE)

ggplot(HR_inhospital) +

geom_line(aes(x=bcr_max, y=yhat), linetype=1, size=1, color="red") +

geom_ribbon(aes(x=bcr_max, ymin=lower, ymax=upper), alpha=0.3, fill="red") +

geom_hline(yintercept=1, linetype=2, size=1) +

annotate("text", x=max(HR_inhospital$bcr_max) * 0.9, y=max(HR_inhospital$upper), label=paste("P for non-linear:<", round(0.001, 4)), hjust=1, vjust=-1) +

geom_vline(xintercept=12.5, linetype=2, linewidth=1, color="black") +

geom_vline(xintercept=22, linetype=2, linewidth=1, color="black") +

theme_classic() +

labs(title="RCS for long-term mortality", x="Blood Urea Nitrogen/Creatinine Ratio", y="Hazard Ratio (95% CI)")

mydata_filtered <- subset(mydata, gender == "Male")

fit <- coxph(Surv(month, status) ~ group , data = mydata_filtered)

summary(fit)

mydata_filtered <- subset(mydata, gender == "Female")

fit <- coxph(Surv(month, status) ~ group , data = mydata_filtered)

summary(fit)

mydata_filtered <- subset(mydata, Age < 65)

fit <- coxph(Surv(month, status) ~ group , data = mydata_filtered)

summary(fit)

mydata_filtered <- subset(mydata, Age >= 65)

fit <- coxph(Surv(month, status) ~ group , data = mydata_filtered)

summary(fit)

mydata_filtered <- subset(mydata, SAPSII < 40)

fit <- coxph(Surv(month, status) ~ group , data = mydata_filtered)

summary(fit)

mydata_filtered <- subset(mydata, SAPSII >= 40)

fit <- coxph(Surv(month, status) ~ group , data = mydata_filtered)

summary(fit)

mydata_filtered <- subset(mydata, SOFA <= 2)

fit <- coxph(Surv(month, status) ~ group , data = mydata_filtered)

summary(fit)

mydata_filtered <- subset(mydata, SOFA > 2)

fit <- coxph(Surv(month, status) ~ group , data = mydata_filtered)

summary(fit)

mydata_filtered <- subset(mydata, elixhauser_vanwalraven <= 11)

fit <- coxph(Surv(month, status) ~ group , data = mydata_filtered)

summary(fit)

mydata_filtered <- subset(mydata, elixhauser_vanwalraven > 11)

fit <- coxph(Surv(month, status) ~ group , data = mydata_filtered)

summary(fit)

mydata_filtered <- subset(mydata, ethnicity == "White")

fit <- coxph(Surv(month, status) ~ group , data = mydata_filtered)

summary(fit)

mydata_filtered <- subset(mydata, ethnicity == "Black")

fit <- coxph(Surv(month, status) ~ group , data = mydata_filtered)

summary(fit)

mydata_filtered <- subset(mydata, ethnicity == "Other")

fit <- coxph(Surv(month, status) ~ group , data = mydata_filtered)

summary(fit)

mydata_filtered <- subset(mydata, BMI < 25)

fit <- coxph(Surv(month, status) ~ group , data = mydata_filtered)

summary(fit)

mydata_filtered <- subset(mydata, BMI >= 25 & BMI < 30)

fit <- coxph(Surv(month, status) ~ group, data = mydata_filtered)

summary(fit)

mydata_filtered <- subset(mydata, BMI >= 30)

fit <- coxph(Surv(month, status) ~ group , data = mydata_filtered)

summary(fit)

analyze_interaction <- function(variable) {

formula_no_interaction <- as.formula(paste("Surv(month, status) ~ group +", variable))

fit_no_interaction <- coxph(formula_no_interaction, data = mydata)

formula_with_interaction <- as.formula(paste("Surv(month, status) ~ group *", variable))

fit_with_interaction <- coxph(formula_with_interaction, data = mydata)

anova_test <- anova(fit_no_interaction, fit_with_interaction, test="Chisq")

return(anova_test)

}

results_Age <- analyze_interaction("Age")

results_gender <- analyze_interaction("gender")

results_ethnicity <- analyze_interaction("ethnicity")

results_BMI <- analyze_interaction("BMI")

results_SAPSII <- analyze_interaction("SAPSII")

results_SOFA <- analyze_interaction("SOFA")

results_ECI <- analyze_interaction("elixhauser_vanwalraven")

print("Age Interaction:")

print(results_Age)

print("Gender Interaction:")

print(results_gender)

print("Ethnicity Interaction:")

print(results_ethnicity)

print("BMI Interaction:")

print(results_BMI)

print("SAPSII Interaction:")

print(results_SAPSII)

print("SOFA Interaction:")

print(results_SOFA)

print("ECI Interaction:")

print(results_ECI)
